# l-Menthol mouth rinse or ice slurry ingestion during the latter stages of exercise in the heat provide a novel stimulus to enhance performance despite elevation in mean body temperature

**DOI:** 10.1007/s00421-018-3970-4

**Published:** 2018-08-20

**Authors:** Owen Jeffries, Matthew Goldsmith, Mark Waldron

**Affiliations:** 1 School of Biomedical Sciences, Faculty of Medical Sciences, Newcastle University, Cookson Building, Newcastle upon Tyne, NE2 4HH UK; 2 School of Sport, Health and Applied Science, St Mary’s University, London, UK; 3 School of Science and Technology, University of New England, Armidale, NSW Australia

**Keywords:** Menthol, Ice slurry, TTE, Heat, Thermoregulation, Perception

## Abstract

**Purpose:**

This study investigated the effects of l-menthol mouth rinse and ice slurry ingestion on time to exhaustion, when administered at the latter stages (~ 85%) of baseline exercise duration in the heat (35 °C).

**Method:**

Ten male participants performed four time to exhaustion (TTE) trials on a cycle ergometer at 70% *W*_max_. In a randomized crossover design, (1) placebo-flavored non-calorific mouth rinse, (2) l-menthol mouth rinse (0.01%), or (3) ice ingestion (1.25 g kg^−1^), was administered at 85% of participants’ baseline TTE. Time to exhaustion, core and skin temperature, heart rate, rating of perceived effort, thermal comfort and thermal sensation were recorded.

**Results:**

From the point of administration at 85% of baseline TTE, exercise time was extended by 1% (placebo, 15 s), 6% (l-menthol, 82 s) and 7% (ice, 108 s), relative to baseline performance (*P* = 0.036), with no difference between l-menthol and ice (*P* > 0.05). Core temperature, skin temperature, and heart rate increased with time but did not differ between conditions (*P* > 0.05). Thermal sensation did not differ significantly but demonstrated a large effect size (*P* = 0.080; $\eta _{{\text{p}}}^{2}$ = 0.260).

**Conclusion:**

These results indicate that both thermally cooling and non-thermally cooling oral stimuli have an equal and immediate behavioral, rather than physiological, influence on exhaustive exercise in the heat.

## Introduction

During exercise in the heat, an increasing thermal load leads to thermo-behavioral adjustments in work rate or reduction in time to exhaustion at a fixed intensity, due to greater perceptual and physiological strain (MacDougall et al. 1974; Galloway and Maughan 1997; González-Alonso et al. 1999; Tatterson et al. 2000; Nybo and Nielsen 2001; Tucker et al. 2004, 2006). Sensory information relating to body temperature is relayed via central and skin thermoreceptors to a thermoregulatory centre in the hypothalamus, which also integrates information from non-thermal sensory receptors (Fortney and Vroman 1985; Gleeson 1998). Behavioral reductions in self-paced exercise in the heat are initially mediated via rise in skin temperature, which alter thermal perception (comfort and sensation) and later by rise in core temperature, which increase cardiovascular strain and perceived exertion (Fortney and Vroman 1985; Flouris and Schlader 2015). This provides evidence that prioritization of afferent signals, most likely based on the type and magnitude, occur under a progressive thermal load. Therefore, we can suppose that thermoregulatory activity occurs in an ordered manner and may be dependent on the magnitude of afferent feedback relayed to the brain.

Cooling interventions during exercise function to either increase the capacity for heat storage or improve thermal sensation, comfort and exertion (Mundel et al. 2006; Lee et al. 2008; Riera et al. 2014; Stevens et al. 2015, 2016; Trong et al. 2015; Bongers et al. 2015; Flood et al. 2017). We have previously shown that an orally administered l-menthol mouth rinse, which elicits non-thermal cooling, extended exercise time at a fixed RPE in the heat (Flood et al. 2017). This has also been supported elsewhere by improved performance during exhaustive exercise (Mündel and Jones 2010; Schlader et al. 2011; Stevens et al. 2015; Stevens and Best 2016). However, in our study, administration of l-menthol was most effective in the early stages of exercise in the heat when both core and skin temperature was low, and was accompanied by a higher self-selected work rate at a fixed RPE of 16. Subsequent administration of l-menthol at 10 min intervals, as thermal load increased, was unable to recover the rate of decline in power output. Therefore, we questioned whether l-menthol’s effects on perceived exertion related to: (1) the early application of a novel, non-thermal cooling stimuli or (2) its efficacy when thermal load was low in the early stages of exercise in the heat. Thermal cooling using ice slurry ingestion (Vanden Hoek et al. 2004; Siegel et al. 2010; Ross et al. 2011) and ice slurry mouth rinsing (Burdon et al. 2013) has also been shown to be effective in improving heat tolerance and extending exercise performance. Whilst the primary objective of ice slurry ingestion is to mediate reduction in core temperature and increase the capacity for heat storage, it also enhances thermal perception via stimulation of thermoreceptors located within oral and abdominal regions (Siegel and Laursen 2012). Indeed, ice slurry mouthwash has been shown to lower the perceptual responses to exercise in the heat and improve time trial performance (Burdon et al. 2013). Given that both l-menthol and ice slurry ingestion enhance cold sensations by acting on thermoreceptors on the oral mucosal surfaces (Eccles 1994), it is possible that both of these interventions have an immediate influence on thermal perception, yet their independent effects have not been investigated.

Considering the evidence that orally administered thermal (ice) and non-thermal (l-menthol) stimuli improve exercise performance in the heat via perceptual mechanisms, it would appear that changes in oral temperature, per se, are not a requirement for the initiation of thermoregulatory behaviour. Rather, afferent signals emanating in the oral cavity are capable behavioral controllers, overriding underlying thermal threats. However, it is not known whether a single novel application of thermal and non-thermal oral cooling can enhance performance when thermal load is high. In addition, our understanding of whether afferent signals emanating from cold receptors in the oral cavity could be deprioritized when faced with a greater bodily threat to thermal homeostasis is unclear.

Therefore, our aims were to investigate the effects of: (1) a non-thermal cooling menthol mouth wash and (2) a thermally cooling ice slurry ingestion on time to exhaustion at a fixed intensity when administered at ~ 85% of the baseline exercise duration. We hypothesised that the delayed administration of l-menthol solution and the ice slurry at 85% of time-to-exhaustion, during a period of high thermal stress, would immediately reduce thermal perception, and improve exercise time compared to placebo.

## Methods

### Participants

Ten non-heat-acclimated males (age 33 ± 9 years; body mass 76.2 ± 6.5 kg; height 179.3 ± 4.6 cm; peak oxygen uptake [$\dot {V}$O_2peak_) 52.4 ± 5.3 ml kg^−1^ min^−1^; maximal aerobic power output (*W*_max_) 371 ± 27 W], with a minimum of 1 year endurance training, volunteered to take part in the study. None of the participants had visited a hot country in the previous 3 months and all testing took place during the months of January–April (average temperatures ranged from 6 to 12 °C). Participants were asked to keep a food diary for 24 h prior to testing and replicate it before each trial and asked to refrain from alcohol, caffeine and strenuous exercise for the 24 h period prior to testing. All participants gave written informed consent. Ethical approval was provided by St Mary’s University ethics committee, which was conducted in accordance with the 1964 Helsinki declaration.

### Study design

Participants visited the laboratory on six separate occasions. All tests were carried out on an electrically braked cycle ergometer (SRM, Julich, Germany) and took place in an environmental chamber (Sporting Edge UK, Basingstoke, UK). During visit one, participants undertook an incremental exercise test to volitional exhaustion in thermoneutral conditions [16 ± 2 °C, 40 ± 8% relative humidity (RH)] to determine $\dot {V}$O_2peak_ and 70% *W*_max_. All subsequent tests were conducted in the heat (35 ± 0.2 °C, 40 ± 0.5% RH). Visit two was a familiarisation time to exhaustion (TTE) on a cycle ergometer at 70% *W*_max_. Visit three was a baseline performance TTE. Visits 4–6 replicated the TTE with an intervention (ice slurry ingestion, menthol mouth rinse, or placebo mouth rinse), administered at 85% of the participants’ baseline TTE, established in visit two, using a randomized crossover design. Randomisation was conducted by generating random numbers for each condition for all participants using online software (Urbaniak and Plous 2015). Participants were blinded to the original hypothesis of the study and informed that the effect of differing mouth rinses on exercise in the heat was being investigated. For each participant, tests were conducted at the same time of day, and experimental trials were separated by a minimum of 72 h to minimise any acclimation effects.

### Experimental procedure

#### Preliminary testing

Participants were familiarised with the cycle ergometer and saddle and handlebar position were recorded and adjusted as required. Participants then completed a 5-min self-selected warm-up prior to completing an incremental ramp test. The test started at 120 W and increased by 5 W every 15 s until volitional exhaustion. Oxygen uptake was measured using breath-by-breath expired air analysis (Jaeger Vyntus CPX, Hoechberg, Germany). Heart rate (HR) was recorded throughout the trial (Polar Team System^®^, Polar UK). $\dot {V}$O_2peak_ was calculated by measuring the highest 30 s average $\dot {V}$O_2_. *W*_max_ was measured as the highest power output recorded during the test.

#### Experimental trials

Following a familiarisation test, visit two involved a baseline TTE at 70% *W*_max_ that was used to anchor performance in the heat. Participants self-selected their pedal cadence during the first familiarisation trial and were instructed to maintain the same cadence for all subsequent trials. We calculated a test re-test reliability of 4.3% coefficient of variation (CV) in preliminary testing (*n* = 8) (Atkinson and Nevill 1998). On visits 36, participants gave a urine sample and had their semi-nude body mass recorded. Hydration status was measured using a refractometer (Pocket Osmochek, Vitech Scientific Ltd, West Sussex, UK), a reading of > 600 mOsm kg H_2_O indicated the start of de-hydration, in which case the participant consumed 500 ml of water and waited 30 min before any testing began. Heart rate (Polar Team System^®^, Polar UK) was recorded throughout the trial and reported every 2 min. A rectal thermometer (Edale Instruments Ltd., Cambridge, UK) was self-inserted 10 cm past the anal sphincter to measure core temperature (*T*_core_) and recorded every 2 min via a scanning thermometer type CDS 1.0 (Edale Instruments Ltd, Cambridge, UK). Skin thermistors (Grant Instruments Ltd., Cambridge, UK) were then attached to four sites on the participants’ right side of the body; upper chest, mid humerus, mid-calf and mid-thigh (Ramanathan 1964). Skin temperature was recorded continuously via a Squirrel data logger (SQ2010, Grant Instruments Ltd., Cambridge, UK) and reported every 2 min. Mean skin temperature (*T*_skin_) was calculated using Ramanathan’s formula (Ramanathan 1964):

$${T_{{\text{skin}}}}=0.3 \times ({T_{{\text{chest}}}}+{\text{ }}{T_{{\text{arm}}}})+0.2 \times ({T_{{\text{thigh}}}}+{\text{ }}{T_{{\text{calf}}}}).$$

Prior to the main experimental test, participants completed a 10 min standardised warm-up in thermoneutral conditions before moving into the environmental heat chamber. Participants were passively warmed in the chamber until their core temperature reached ~ 37.5 °C. The experimental trial started cycling at 70% of *W*_max_ until volitional exhaustion which was defined as a 10% drop in cadence for longer than 5 s, or if core temperature exceeded 39.5 °C. At 85% of participants’ baseline TTE, one of three interventions were administered: (1) placebo mouth rinse, (2) l-menthol mouth rinse, or (3) ice slurry. Time to exhaustion was recorded and performance from the point administration was also observed. A blood sample via capillary puncture was taken at exhaustion for blood lactate (BLa) analysis (Biosen C-Line, EKF Diagnostics, Germany). Participants towel dried to remove any residual sweat and were weighed to assess changes in semi-nude (cycling shorts only) body mass to estimate sweat loss (kg h^−1^) (Baker et al. 2009). These data were adjusted for fluid intake during the ice slush ingestion trial.

#### Perceptual measurements

Ratings of perceived exertion (RPE) was recorded on a 6–20 point Borg scale (Borg 1982). Thermal comfort (TC) was recorded on a 7-point scale where − 3 = “much too cool”, 0 = “comfortable”, and 3 = “much too warm” (Bedford 1936). Thermal sensation (TS) was recorded on a 9-point scale where − 4 = “very cold”, 0 = “neutral”, and 4 = “very hot” (Zhang et al. 2004). RPE, TC and TS were recorded at: rest; every 5 min during the trial; 10 s prior to administration of the intervention (Pre), 10 s following the intervention (Post) and at exhaustion (End).

#### Drink formulation

l-Menthol solution was formulated from menthol crystals (House of Flavours, Gloucestershire, UK) dissolved in de-ionized water heated to 40 °C at a concentration of 0.64 mM (0.01%). The solution was then cooled and stored at 5 °C for up to 2 months. Prior to use, solutions were aliquoted for mouth rinse (25 ml) and warmed to 19.5 ± 0.5 °C. Ice slurry was made by adding crushed ice to water and mixing in a blender (NutriBullet, Los Angeles, USA), until consistency reached that of an ice slurry (0.3 ± 0.3 °C) and administered 1.25 g kg^−1^ (95 ± 8 g) (Siegel et al. 2011). Placebo was a neutral raspberry flavor non-calorific mouth rinse (25 ml) (FlavDrops, MyProtein, Norwich, UK) (19.3 ± 0.4 °C). The participants either ingested the ice slurry over a period of ~ 10 s or swilled the menthol/placebo mouth rinse for 5 s before spitting the solution into a cup.

### Data analysis

Statistical analyses were performed using SPSS (IBM SPSS statistics 22 Inc, USA) and statistical significance was set at *P* < 0.05. Single time point data was examined for within-group effects across condition using a one-way repeated measures analysis of variance (ANOVA). A two-way repeated measures ANOVA was used to test for within-group effects across condition and time. Where sphericity could not be assumed, a Greenhouse–Geisser correction was applied. Differences in main effects (condition or time) were further analyzed using pairwise comparisons, incorporating a Bonferroni adjustment. Magnitude of effect was calculated with partial eta-squared ($\eta _{{\text{p}}}^{2}$) according to the following criteria: 0.02, a small difference; 0.13, a moderate difference; 0.26 a large difference (Cohen 1988). Data are presented as mean ± SD (*n* = 10).

## Results

**Fig. 1:**
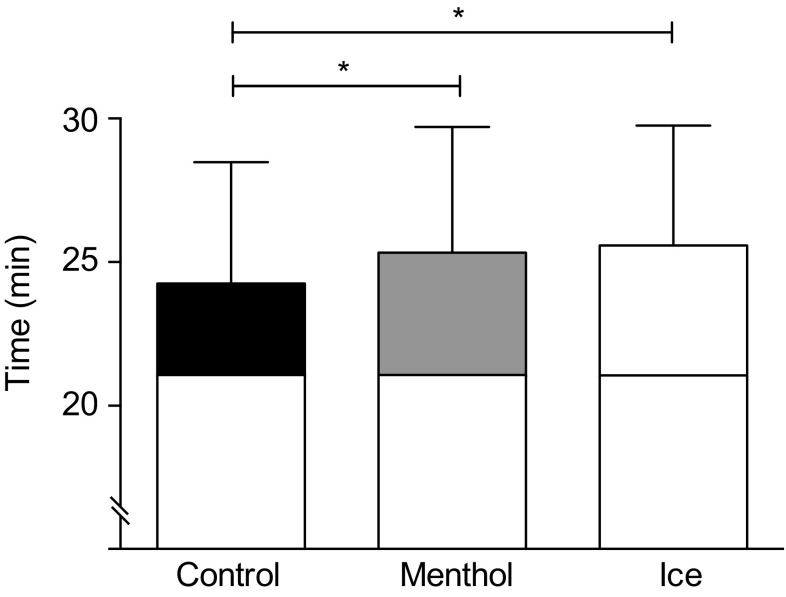
Time to exhaustion following administration of placebo, menthol and ice conditions at 85% of baseline time to exhaustion. Exercise time from the point of administration (21.02 ± 3.53 min) is shown, placebo (black), menthol mouth rinse (gray) ice slurry ingestion (white). All data are shown as mean ± SD, (*n* = 10)

TTE differed between condition [*F*_(2,18)_ = 6.852, *P* = 0.006; $\eta _{{\text{p}}}^{2}$ = 0.432]. Pairwise analysis confirmed that when compared to a placebo-flavored mouth rinse (19 °C) (24:27 ± 4.22 min) exercise time was increased following menthol (25:34 ± 4.37 min; *P* = 0.036) and ice (25:59 ± 4.16 min; *P* = 0.04) with no difference between ice slurry and menthol (*P* > 0.05) in the heat (35 °C) (Fig. 1). From the point of administration at 85% of TTE in trial 1 (21.02 ± 3.53 min), participants exercised for an additional 3:25 ± 1.55 min (placebo), 4:32 ± 2.29 min (menthol) and 4:57 ± 1.27 min (ice), representing a 1% (15 s), 6% (82 s) and 7% (107 s) increase in performance time for placebo, menthol and ice slurry, respectively, relative to baseline performance.

There was no trial order effect (*P* > 0.05), and no significant difference between conditions at the point of drink administration (Table 1): *T*_core_ [*F*_(2,18)_ = 0.184, *P* = 0.834, $\eta _{{\text{p}}}^{2}$ = 0.020]; *T*_skin_ [*F*_(2,18)_ = 0.265, *P* = 0.770, $\eta _{{\text{p}}}^{2}$ = 0.029]; HR [*F*_(2,18)_ = 0.428, *P* = 0.658, $\eta _{{\text{p}}}^{2}$ = 0.045]; TC [*F*_(2,18)_ = 0.310, *P* = 0.737, $\eta _{{\text{p}}}^{2}$ = 0.033]; TS [*F*_(2,18)_ = 0.231, *P* = 0.796, $\eta _{{\text{p}}}^{2}$ = 0.025]; RPE [*F*_(2,18)_ = 0.448, *P* = 0.646, $\eta _{{\text{p}}}^{2}$ = 0.047].

**Table 1:** Physiological and perceptual values at the point of drink administration for placebo, menthol and ice slurry conditions

	Placebo	Menthol	Ice slurry
*T*_core_ (°C)	38.5 ± 0.3	38.5 ± 0.2	38.4 ± 0.3
MST (°C)	35.5 ± 0.7	35.5 ± 1.0	35.7 ± 0.7
HR (beats/min)	178.2 ± 12.0	176.7 ± 9.4	177.8 ± 8.7
TC (− 3 to 3)	2.3 ± 0.7	2.2 ± 0.8	2.3 ± 0.7
TS (− 4 to 4)	3.1 ± 0.8	3.0 ± 1.1	3.1 ± 1.1
RPE (6–20)	17.3 ± 1.9	17.1 ± 2.2	17.3 ± 2.0

All data are shown as mean ± SD (*n* = 10)

${T_{core}}$ core temperature, *MSK* mean skin temperature, *HR* heart rate, *TC* thermal comfort, *TS* thermal sensation, *RPE* rating of perceived exertion

**Fig. 2:**
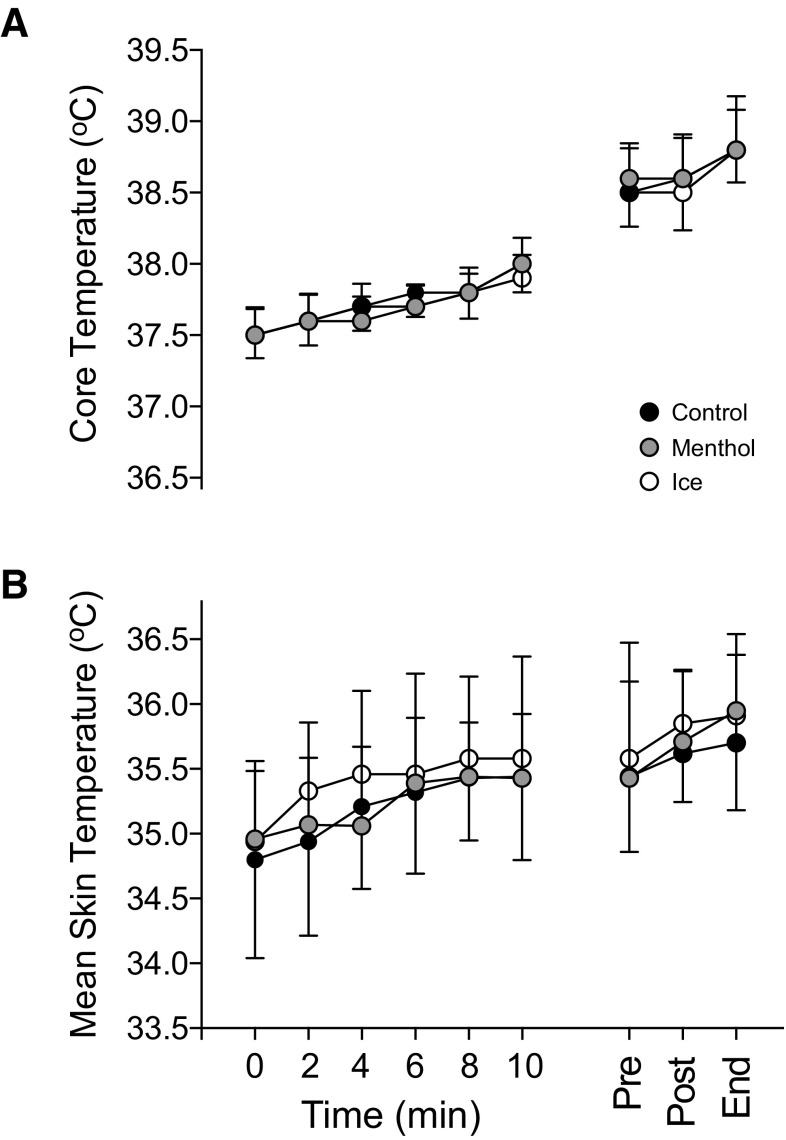
**a** Core temperature (°C) and **b** mean skin temperature (°C) following administration of placebo, menthol and ice slurry conditions at 85% of baseline time to exhaustion. Placebo (black), menthol mouth rinse (gray) ice slurry ingestion (white). All data are shown as mean ± SD, (*n* = 10)

Core temperature was similar at the beginning of the trial (placebo: 37.5 ± 0.2 °C; menthol: 37.5 ± 0.2 °C; ice: 37.6 ± 0.2 °C) and increased with time [*F*_(8,72)_ = 141.421, *P* < 0.001; $\eta _{{\text{p}}}^{2}$ = 0.940]; however, there was no difference between conditions [*F*_(2,18)_ = 0.161, *P* = 0.852; $\eta _{{\text{p}}}^{2}$ = 0.018] (Fig. 2a). End core temperature was not different between conditions (placebo: 38.9 ± 0.4 °C; menthol: 38.8 ± 0.3 °C; ice slurry: 38.7 ± 0.3 °C). Mean skin temperature increased with time [*F*_(8,72)_ = 31.495, *P* < 0.001; $\eta _{{\text{p}}}^{2}$ = 0.778]; however, there was no difference between conditions [*F*_(2,18)_ = 0.914, *P* = 0.359; $\eta _{{\text{p}}}^{2}$ = 0.107] (Fig. 2b).

**Fig. 3:**
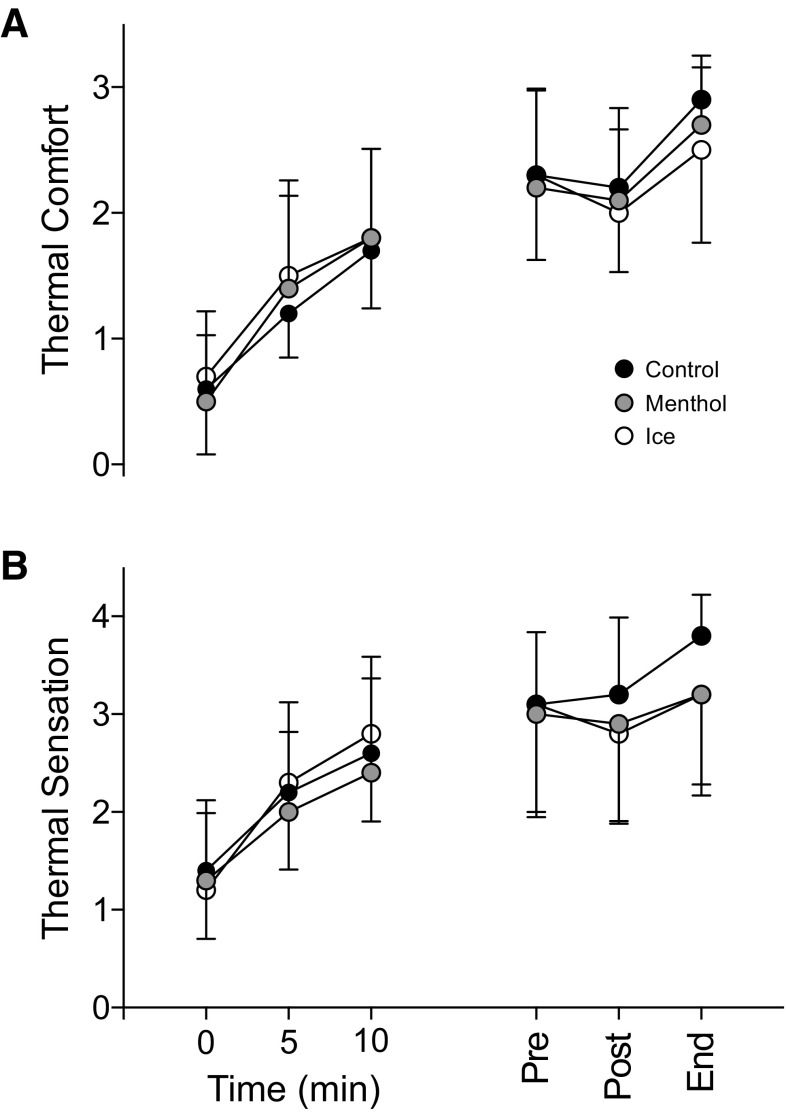
**a** Rating of thermal comfort and **b** thermal sensation following administration of placebo, menthol and ice slurry conditions at 85% of baseline time to exhaustion. Placebo (black), menthol mouth rinse (gray) ice slurry ingestion (white). All data are shown as mean ± SD, (*n* = 10)

Thermal comfort increased with time [*F*_(5,45)_ = 58.857, *P* < 0.001; $\eta _{{\text{p}}}^{2}$ = 0.867]; however, there was no difference between conditions [*F*_(2,18)_ = 0.060, *P* = 0.942; $\eta _{{\text{p}}}^{2}$ = 0.007] (Fig. 3a). Thermal sensation increased with time [*F*_(5,45)_ = 30.298, *P* < 0.001; $\eta _{{\text{p}}}^{2}$ = 0.771]. There was no difference between conditions [*F*_(2,18)_ = 2.909, *P* = 0.080; $\eta _{{\text{p}}}^{2}$ = 0.260]; however, there were large effect sizes between conditions, suggesting that thermal sensation may have been reduced (Fig. 3b).

RPE increased with time [*F*_(2.638,23.740)_ = 184.914, *P* < 0.001; $\eta _{{\text{p}}}^{2}$ = 0.954]; however, there was no difference between conditions [*F*_(2,18)_ = 0.404, *P* = 0.674; $\eta _{{\text{p}}}^{2}$ = 0.043]. End test blood lactate was not different between conditions [*F*_(2,18)_ = 0.244, *P* = 0.786; $\eta _{{\text{p}}}^{2}$ = 0.026]. Heart rate increased with time [*F*_(8,72)_ = 309.647, *P* < 0.001; $\eta _{{\text{p}}}^{2}$ = 0.972]; however, there was no difference between conditions [*F*_(2,18)_ = 0.840, *P* = 0.448; $\eta _{{\text{p}}}^{2}$ = 0.085]. Body mass was reduced between pre- and post-trial [*F*_(1,9)_ = 141.525, *P* < 0.001; $\eta _{{\text{p}}}^{2}$ = 0.940]; however, this was not different between conditions [*F*_(2,18)_ = 1.756, *P* = 0.201; $\eta _{{\text{p}}}^{2}$ = 0.163].

## Discussion

We investigated the effects of l-menthol mouth rinse and ice slurry ingestion on time to exhaustion, when administered at the latter stages (~ 85%) of baseline exercise duration in the heat (35 °C). Our main finding was that thermal and non-thermal cooling of the oral cavity using l-menthol mouth rinse or ice slurry, respectively, increased total TTE by ~ 6% (82 s) and 7% (107 s), respectively, compared to baseline performance. These changes were larger than the typical error of the TTE (CV% = 4.3), indicating that a real change in performance was observed. The ergogenic effects of the cooling strategies were apparent in the absence of any change body temperature or other physiological variables. Similarly, there were no significant changes in thermal comfort or thermal sensation; however, there were large effect sizes noted for thermal sensation between the two cooling conditions and placebo, inferring the presence of a perceptual cooling effect. Collectively, these findings demonstrate that both thermally cooling and non-thermally cooling oral stimuli have an equal and immediate behavioral, rather than physiological, influence on exhaustive exercise in the heat.

Our findings confirm, and expand upon, recent work investigating oral mouth rinsing with l-menthol. Oral l-menthol has typically been intermittently administered (3–6 times) over the course of an endurance exercise bout (Mündel and Jones 2010; Riera et al. 2014; Stevens et al. 2015; Trong et al. 2015; Flood et al. 2017). To our knowledge, this was the first study to intervene during a period of advanced thermal stress by administering orally a single ice slurry or menthol mouth rinse, with the aim of rapidly altering thermal perception of the athlete. The immediate effects elicited by both cooling strategies in the current study adds to the extant literature by demonstrating that: (1) the psychophysical effects of l-menthol appear to be at least equal to that of ice slurry; (2) these effects occur with immediate effect on physical performance and (3) both of these interventions are capable of overriding the deleterious effects experienced during baseline performance, presumably elicited via a combination of afferent cues (i.e., internal thermal and metabolic perturbations).

We previously speculated that the effects of oral l-menthol on perceived exertion and thermal sensation might dissipate as a function of exercising heat exposure (Flood et al. 2017). We suggested that afferent cues from the oral cavity may be deprioritized when homoeostasis is challenged through increased core and skin temperature. The current findings would entirely refute our previous supposition. During periods of progressive thermal stress, imposed by the combination of exercise and environmental heat and humidity, both l-menthol and ice slurry ingestion offered an immediate cooling stimulus. Indeed, at the point of administration, core temperature (~ 38.5 °C) and mean skin temperature (~ 35.6 °C) were increased compared to the start of exercise. We also observed an immediate reduction in thermal sensation with no change in thermal comfort following administration of menthol and ice slurry. Thermal comfort is described to reflect the state of mind that expresses satisfaction with the surrounding environment, whilst thermal sensation results from the perception of stimulus generated by peripheral and central thermosensors (Flouris 2011). It would appear that the cold sensations, emanating in the oral cavity, have the capacity to influence the control of exercise intensity by overriding the underlying, yet progressively changing thermal threats and inducing immediate behavioral adjustments. The importance of perceptual cooling is reinforced by the limited effects of ice slurry ingestion following exercise-induced hyperthermia (39.3 °C) on absolute measures of voluntary activation or muscle force production reported elsewhere (Burdon et al. 2014).

Both of these thermo-effective interventions have potential to act upon the transient receptor potential (TRP) family of oral mucosal receptors, which relay information to the brain regarding the perception of temperature (McKemy et al. 2002; Peier et al. 2002; Knowlton and McKemy 2011; Andersen et al. 2014). Here, the homeostatic set-point error can be determined and iteratively acted upon, in combination with the milieu of other feedback loops (St Clair Gibson et al. 2018). In concert with other peripheral feedback mechanisms (Lambert et al. 2005), these sub-conscious cues facilitate the conscious behavioral and subsequent physiological adjustments that are necessary to protect bodily homeostasis (i.e., thermal balance) from catastrophic derangement. Cold and menthol-induced cold sensations are thought to primarily be transduced by the TRPM8 voltage-gated ion channel present on Aδ and C-sensory nerve fibers (McKemy et al. 2002). A recent review on non-thermal cooling interventions suggested that menthol application could also inhibit the TRPA1 channel, thereby mediating pain responses and reducing a possible ergolytic influence of pain sensations (Stevens et al. 2018). While it is possible that pain is inhibited, multimodal signaling can occur in somatosensory neurons whereby fibers expressing TRPM8 relay information to both thermal and nociceptive pathways (Belmonte and Viana 2008; Green and Akirav 2010). Therefore, it is feasible that the actions of l-menthol are more complex than previously postulated in the sports performance literature and that cooling sensations conferred to the athlete are, perhaps, co-joined with ‘distractors’ from the stressful thermal and physiological cues. During simple repetitive tasks, such as cycling, it may be advantageous to engage in dissociative strategies (Bigliassi et al. 2017), reallocating attention towards novel (and possibly moderately painful) stimuli, permitting background projections of thermal or metabolic cues and lower ‘weighting’ of their overall influence (St Clair Gibson et al. 2018). Clearly, further research is necessary to explore the above suggestions.

It was previously reported that ice slurry pre-exercise ingestion did not alter thermal sensation, despite lowering core temperature (Stevens et al. 2015). In the same trial, oral l-menthol lowered thermal sensation and extended time to exhaustion compared to the ice condition. The differences to the ice condition in our study are partly explained by the timing of the ingestion but one would anticipate the ingestion of ice to provide a psychophysical effect. For example, ice slurry ingestion is thought to stimulate thermoreceptors in oral and abdominal regions (Siegel and Laursen 2012), as well as the reward/pleasure centres of the brain, leading to an increase in central drive and motivation (Guest et al. 2007). Guest et al. (2007) introduced different temperatures of artificial saliva into the mouth and recorded activation of brain regions and perceived pleasantness. The authors found that a cold fluid (5 °C) was perceived to be more pleasant when compared to a warm (50 °C) solution and that some of the same brain regions involved in detecting temperature were involved in sensing pleasantness. Therefore, it is possible that pleasant stimuli helped to maintain central drive and increase motivation for exercise, partly explaining the reason for a longer exercise duration with ice slurry (~ 0.3 °C) compared to placebo (~ 19.5 °C).

Our findings have some potential implications for athletes who compete in endurance events. Based on the current preliminary evidence, it is feasible that a single administration of l-menthol or ice slurry would elicit an almost identical effect on exercise capacity, without conferring a notable physiological change. These effects are, therefore, likely to be based on an alteration in the sensation and subsequent perception of the thermal load. While both strategies elicited similar performance effects, the l-menthol administration is the most practical choice and could be carried about the person during competition or training. However, further research is needed to corroborate these preliminary findings and explore the potential magnitude of these effects in practical scenarios prior to any field application.

## Conclusion

In summary, non-thermal cooling (l-menthol) and thermal cooling (ice slurry) of the oral cavity when administered at the latter stages (~ 85%) of baseline exercise duration in the heat (35 °C) are capable of extending exercise performance. This occurs in the absence of any changes in body temperature or other physiological variables. The observed reduction in thermal sensation suggests that the mechanism may relate to a diminished perception of heat stress, enhanced motivation or distraction from stressful thermal and physiological cues, thereby enhancing performance.

## Abbreviations

HR: Heart rate
MST: Mean skin temperature
RPE: Rating of perceived exertion
TC: Thermal comfort
*T*_(core)_: Core temperature
TS: Thermal sensation
*T*_(skin)_: Skin temperature
TTE: Time to exhaustion
$\dot {V}$O_2peak_: Peak oxygen uptake
*W*_max_: Maximum power output achieved at $\dot {V}$O_2peak_
